# Is right ventricular outflow tract epicardial substrate ablation the standard of care in high-risk Brugada syndrome?

**DOI:** 10.1093/europace/euae020

**Published:** 2024-01-22

**Authors:** Elijah R Behr, Giulio Conte, Arthur Wilde

**Affiliations:** Cardiovascular and Genomics Research Institute, St George’s, University of London, Cranmer Terrace, London SW17 0RE, UK; Cardiology Care Group, St George’s University Hospitals NHS Foundation Trust, London SW17 0QT, UK; Mayo Clinic Healthcare, London W1B 1PT, UK; Electrophysiology Unit, Department of Cardiology, Fondazione Cardiocentro Ticino, via Tesserete 48, Lugano, Switzerland; Department of Cardiology, Amsterdam UMC, University of Amsterdam, Amsterdam, The Netherlands; Amsterdam Cardiovascular Sciences, Heart Failure and Arrhythmias, Amsterdam, The Netherlands; European Reference Network for Rare, Low Prevalence and Complex Diseases of the Heart (ERN GUARD-Heart)

**This editorial refers to ‘High-risk Brugada syndrome: factors associated with arrhythmia recurrence and benefits of epicardial ablation in addition to implantable cardioverter defibrillator implantation’ by V. Santinelli *et al.*, https://doi.org/10.1093/eupace/euae019.**

The most recent ESC guidelines for the management of patients with ventricular arrhythmias and the prevention of sudden cardiac death, published in 2022^1^ indicate that radiofrequency catheter ablation (RFA) should be considered for Brugada syndrome (BrS) patients with recurrent implantable cardioverter defibrillator (ICD) shocks despite medical therapy. These recommendations were based on data from various series from different centres, starting with Haissaguerre and colleagues reporting the elimination of triggering ectopic beats, followed by seminal work from Nademanee and colleagues, reporting right ventricular outflow tract (RVOT) epicardial substrate ablation.^2^ The substrate has been shown to be formed by subepicardial myocardial fibrosis, suggesting an underlying subepicardial cardiomyopathy.^3^ Publications prior to and since these guidelines have offered supporting evidence (see *Table 1*), all having shown benefit in reduction in shock frequency in this high-risk patient group. The most notable of these was the BRAVO registry published last year, reporting on the outcomes in 159 high-risk BrS patients treated at specialist centres, of whom 140 (88%) had experienced numerous ICD shocks for ventricular fibrillation (VF). The outcomes were excellent, with 81% being completely VF free after one procedure and 96% after a further procedure.^8^

**Table 1:** Recent ablation studies reporting independent populations of BrS patients

Study	Number of patients	Indications	Ablation strategy	Follow-up time (months)	Survival free from arrhythmic events (%)	Major complication rate (%)
Pappone *et al*.^4^	135	Spontaneous VT/VF	RVOT epicardial substrate ablation	10 (median)	98.5	0
		OR				
		symptoms AND inducible VT/VF				
Talib *et al*.^5^	21	Drug-resistant VF	PVC trigger ablation	56 ± 36 (mean)	67	0
		OR	AND			
		electrical storm	RV/RVOT endocardial substrate ablation			
Salghetti *et al*.^6^	36	Symptomatic PVCs	Hybrid thoracoscopic RVOT epicardial ablation	16 ± 8 (mean)	77.8	2.8 (cardiac tamponade)
		OR				
		ICD shocks				
		OR				
		documented VT/VF				
Li *et al*.^7^	18	Symptomatic AND refusing an ICD	RV/RVOT endocardial and epicardial substrate ablation	46.2 (median)	94	5.6 (cardiac tamponade)
			OR			
			PVC trigger ablation			
Nademanee *et al*.^8^	159	Spontaneous VF with or without frequent shocks	Epicardial substrate ablation	48 ± 29 (mean)	81 (single procedure)	2.5 (haemo-pericardium)
					96 (repeat procedure)	
Santinelli *et al*.^9^	206	ACA	RVOT only epicardial substrate ablation	40 (median)	99.5	0
		OR				
		‘malignant syncope’				

ACA, aborted cardiac arrest; BrS, Brugada syndrome; ICD, implantable cardioverter defibrillator; PVC, premature ventricular complex; RV, right ventricle; RVOT, RV outflow tract; VF, ventricular fibrillation; VT, ventricular tachycardia.

This and other studies indicated the importance of using the sodium channel blocker challenge to unveil the true extent of the RVOT substrate, and its nature, i.e. delayed and discontinuous conduction. Thus, amelioration and elimination of the Type 1 Brugada electrocardiogram pattern even when the sodium channel blocker is administered appears to represent the most desirable endpoint for success.^4,8,10^ Indeed, a major reason identified for recurrence of ventricular tachycardia (VT)/VF was incomplete ablation of the substrate due to early studies not employing sodium channel blockers during the procedure.^8^ Protocols have also used the elimination of the inducibility of polymorphic VT or VF by programmed electrical stimulation as an endpoint.

Another feature associated with recurrence was the presence of the early repolarization pattern (ERP). Patients with BrS and ERP were found to have a more extensive substrate including the RVOT and inferior right ventricular or inferolateral left ventricular walls.^11^

## Radiofrequency catheter ablation beyond aborted cardiac arrest and prior implantable cardioverter defibrillator shocks?

From 2015 to 2016, research into epicardial RFA from the group led by Pappone *et al.*^4^ included symptomatic BrS patients without prior aborted cardiac arrest (ACA) or recurrent ICD shocks. Only 27 of 135 enrolled patients had suffered prior ICD therapy, while 72 had not suffered prior VT/VF, but VT/VF was inducible at programmed electrical stimulation. Ajmaline was used during epicardial mapping and outcomes were excellent without acute complications and only two recurrences.

In this context, Santinelli *et al*.^9^ build on their previous work with a report of a cohort of 257 high-risk BrS patients, collected since 2017, who presented with ‘malignant syncope’ (176) or ACA (81). All had undergone ICD implantation and, if they had suffered an ACA, had demonstrated a negative coronary angiogram. If they had presented with syncope without documented VT/VF, they had undergone an unspecified diagnostic work-up that included a tilt table test. The cohort was selected from a larger group of 755 patients also described as high risk, although the characteristics of the denominator population and the sub-selection process were not declared.

The initial period between ICD implantation and RFA was used to analyse the predictors of cardiac events [sudden cardiac death (SCD), ACA, and ICD shocks]. Subsequently, 206 patients underwent epicardial mapping and RFA and 51 patients declined RFA and were used as a comparator group, i.e. RFA vs. no-RFA. Independent risk factors for cardiac events included substrate size, prior ACA, or hosting a *SCN5A* genetic variant. In the post-RFA follow-up (median 40 months), the RFA group demonstrated superior outcomes compared with no-RFA, with only one RFA patient suffering a recurrence compared with 15 subjects in the no-RFA group.

Patients with the ERP and therefore a more extensive substrate were excluded from the study, which may explain the higher success rates than the BRAVO registry (*Table 1*) that included this patient group. The BRAVO registry also showed a 2.5% rate of haemopericardium, while Santinelli *et al*. reported a remarkable absence of any serious adverse event. Furthermore, Santinelli *et al*. used ajmaline systematically in a high-risk cohort, many of whom hosted pathogenic *SCN5A* variants. This can place patients at high risk for proarrhythmia and has even resulted in a patient requiring Extra Corporeal Membrane Oxygenation, as reported by the Pappone and Santinelli lab.^12,13^ Given that major procedural complication rates reported in *Table 1* range from 0 to 5.6%, it seems unlikely that such low levels of serious adverse events are likely to be replicated if epicardial RFA is more commonplace.

Importantly, the cohort reported by Santinelli *et al*.^9^ is not based on individuals with recurrent ICD shocks. Specifically, the majority of patients included had originally presented with syncope rather than ACA and only 26% went on to have shocks, of whom about half had multiple shocks.

Syncope patients are not as high a risk group as those who have experienced prior ACA or prior appropriate ICD shock therapy. The differentiation of truly arrhythmic syncope from other forms of loss of consciousness is challenging and patients with a range of risk are often included. Thus, large-scale multi-centre studies of BrS patients have indicated lower cardiac event rates in patients presenting with syncope compared with those with ACA. Indeed, the combined SCD, ACA, and ICD shock annual event rates in the FINGER study were 1.9% in patients who first presented with syncope and 7.7% in those who first presented with ACA.^14^

In the report by Santinelli *et al*.,^9^ over a median of 27 months follow-up, 22 of 141 patients with initial syncope experienced ICD shocks prior to RFA, suggesting an ∼7% annual event rate. A total of 5 out of 35 subjects experienced an ICD shock in the no-RFA group prior to declining RFA over a 26-month median follow-up, suggesting an ∼6.6% annual event rate. This indicates that the patients with syncope who were included in their study are not typical of most BrS patients with syncope but are a highly selected group, potentially biased by the selection process that led to their inclusion in the study cohort. However, relying on ICD shocks as the surrogate for life-threatening events may have led to an overestimation of the true risk for sustained VF and SCD.

The aforementioned ESC guidelines^1^ also indicate that medical therapy should be attempted prior to ablation therapy, and indeed, the use of quinidine or hydroquinidine precedes the use of ablation therapy in the management pathway. In Santinelli’s study, the intolerance to these medications was extremely high, with 53 of 79 patients in the RFA group having to discontinue treatment. Much lower rates have been described by other centres.^15^

## What do we need before indications for radiofrequency catheter ablation include syncope?

Additional evidence beyond a single centre experience will therefore be necessary before ‘epicardial ablation guided by ajmaline administration’ can be endorsed as ‘a safe and more effective strategy to prevent VF events and VF storms’ in patients with ‘high-risk’ BrS as defined in this study, and thus expanding the use of RFA to patients who present with syncope. The risk of adverse events from epicardial ablation and ajmaline administration requires balancing with the potential benefit in a randomized case–control study where a comparison with hydroquinidine and quinidine would also seem appropriate. Furthermore, a systematic and evidence-based approach to evaluation of syncope will be required to ensure the inclusion of appropriate symptomatic patients. Of course, the use of RFA in asymptomatic patients remains a Class III recommendation with no supportive data.

Thus, on the current evidence base, epicardial RVOT substrate ablation is **not** suitable for all ‘high-risk’ patients with BrS, especially those with syncope.
